# Population pharmacokinetics of fosmidomycin and clindamycin in combination with artesunate for uncomplicated *Plasmodium falciparum* malaria in Gabonese children and adults

**DOI:** 10.1186/s12936-026-05872-6

**Published:** 2026-03-19

**Authors:** Christoph Pfaffendorf, Jean Claude Dejon-Agobé, Jean Ronald Edoa, Oumou Maïga-Ascofaré, Ebenezer Ahenkan, Ayôla Akim Adegnika, Michael Ramharter, Sebastian G. Wicha, Johannes Mischlinger

**Affiliations:** 1 Department of Clinical Pharmacy, Institute of Pharmacy, University of Hamburg, Bundesstrasse 45, 20146 Hamburg, Germany; 2 Center for Tropical Medicine, Bernhard Nocht Institute for Tropical Medicine and I., Department of Medicine, University Medical Center Hamburg-Eppendorf, Hamburg, Germany; 3 German Centre for Infection Research, Partner Site Hamburg-Lübeck-Borstel-Riems, Hamburg, Germany; 4 Centre de Recherches Médicales de Lambaréné, Lambaréné, Gabon; 5 Kumasi Centre for Collaborative Research in Tropical Medicine, Kumasi, Ghana; 6 Infectious Disease Epidemiology, Bernhard Nocht Institute for Tropical Medicine, Hamburg, Germany; 7 Department of Implementation Research, Bernhard Nocht Institute for Tropical Medicine, Hamburg, Germany; 8 Institut für Tropenmedizin, Eberhard-Karls-Universität Tübingen, Tübingen, Germany; 9 German Centre for Infection Research (DZIF), Tübingen, Germany; 10 Department of Pharmacology, Kwame Nkrumah University of Science and Technology, Kumasi, Ghana

**Keywords:** Fosmidomycin, Clindamycin, Pharmacometrics, Pharmacokinetics, Malaria

## Abstract

**Background:**

The increasing prevalence of artemisinin resistance in *Plasmodium falciparum* malaria highlights the urgent need for new, effective treatment strategies. The combination of fosmidomycin and clindamycin has shown promise, but further investigation is required, particularly regarding its pharmacokinetics. This study aimed to develop population pharmacokinetic models for fosmidomycin and clindamycin administered alongside artesunate, to evaluate the adequacy of weight-based dosing in children and adults with uncomplicated malaria and to identify relevant covariates influencing drug disposition.

**Methods:**

This analysis was conducted within an open-label, randomized clinical trial in Lambaréné, Gabon. Forty patients aged 3–57 years with uncomplicated *P. falciparum* malaria received an oral combination of artesunate (2 mg/kg), fosmidomycin (30 mg/kg), and clindamycin (10 mg/kg) administered every 12 h for three days. Plasma samples were collected at various time points and analyzed via LC–MS/MS. Population pharmacokinetic models for fosmidomycin and clindamycin were developed using non-linear mixed-effects modeling to characterize drug exposure and identify significant covariates.

**Results:**

Pharmacokinetic data were best described by one-compartment models with first-order absorption for both drugs. Allometric scaling based on body weight was a significant covariate influencing clearance and volume of distribution for both fosmidomycin and clindamycin. Notably, body temperature was identified as a significant covariate affecting fosmidomycin clearance. Dosing based on body weight resulted in consistent exposure (AUCτ) of fosmidomycin across all age groups. However, clindamycin exposure was substantially lower in children under 12 years of age compared to older patients.

**Conclusion:**

We successfully developed robust population pharmacokinetic models for both fosmidomycin and clindamycin. These models confirmed that body weight is a key determinant of drug disposition for both agents, and that body temperature influences fosmidomycin clearance. While the current fosmidomycin dosing appears adequate across age groups, the standard 10 mg/kg dose of clindamycin results in lower exposure in young children compared to adolescence and adults. Dose simulation suggests that increasing clindamycin to 12 mg/kg in children under 35 kg would provide equal drug exposure in all age groups.

**Supplementary Information:**

The online version contains supplementary material available at 10.1186/s12936-026-05872-6.

## Background

With an estimated 263 million cases and 597,000 deaths in 2023, malaria continues to be one of the most significant challenges in global health. Alongside vector control and new vaccinations efforts, efficacious and safe pharmacotherapy is crucial in combating malaria-related mortality worldwide [1]. Artemisinin-based combination therapies (ACTs), introduced at the turn of the millennium, have become the first-line treatment recommended by the World Health Organization (WHO) for all malaria-endemic countries [2, 3]. However, over the past 15 years, the efficacy of ACTs has diminished in the Greater Mekong region in Southeast Asia. This decline is largely due to the spread of parasites with increased artemisinin tolerance caused by mutations in the Pf*kelch13* gene [4]. Coupled with the emergence of resistance to commonly used partner drugs, the cure rates of ACTs dropped below acceptable levels in the region [1, 4]. Recent years have also seen the first indications of increased artemisinin tolerance emerging in sub-Saharan Africa. A continuation of this trend could jeopardize the use of ACTs as the first-line treatment in these regions, posing a significant threat to ongoing malaria control efforts [1, 5, 6]. Therefore, innovation is needed in the treatment of *Plasmodium falciparum* infections.

A suggested reason for the development of resistance to the ACTs is the pharmacokinetic mismatch between the artemisinin derivatives and the partner drugs. Artemisinin derivates all have a short elimination half-live of below 4 h while the partner drugs show long elimination half-lives of several days up to 4 weeks [7]. Therefore, the drugs can only protect each other for a short period of time after which the partner drug remains in the body for a long time at sub-therapeutic levels.

One novel option for the treatment of malaria is the drug fosmidomycin. It was initially discovered in the 1980s and was developed as an antibiotic for urinary tract infections due to its supposed similarity to fosfomycin. Despite promising early results, its development was halted following early Phase I and II trials, most likely due to the development of more promising antibiotics during the same time [8–10]. In the 2000s, it was determined that fosmidomycin has a distinct mechanism of action from fosfomycin and was, in fact, not inhibiting the cell wall synthesis but acting as an inhibitor of a crucial step in the isoprenoid synthesis of bacteria and parasites using the non-mevalonate pathway. Over the subsequent decades, fosmidomycin was explored in various studies for the treatment of malaria, both as a monotherapy and in combination with other drugs. Notably, the lincosamide antibiotic clindamycin was found to exert a synergistic effect when combined with fosmidomycin. However, these studies assessing 3 days regimens did not reliably achieve the 95% threshold for cure rates recommended by the WHO, although some results were close [11].

Fosmidomycin was included in the MultiMal study, a phase II clinical trial assessing new combination therapies for uncomplicated malaria in children and adults. This trial aimed to compare these new combinations against the standard of care ACTs, with a particular emphasis on the pharmacokinetics of the different compounds. The clinical outcomes of this trial are published separately.

Among the combinations tested, fosmidomycin and clindamycin were combined with artesunate. The present publication focuses on the population pharmacokinetics of fosmidomycin and clindamycin, particularly examining the weight-based dosing regimens and their exposure profiles in different age groups.

## Patients and methods

### Study design

This analysis was part of an open label randomized controlled clinical phase II trial investigating the pharmacokinetics, efficacy and tolerability of two novel antimalarial combination therapies (artesunate-pyronaridine-atovaquone/proguanil & artesunate-fosmidomycin-clindamycin) compared to a standard ACT (artesunate-pyronaridine). The study was conducted in Lambaréné, Gabon, and Kumasi, Ghana for organizational reasons the arm containing fosmidomycin and clindamycin was only conducted in Lambaréné, Gabon. A weight-based dosing regimen of an artesunate-clindamycin-fosmidomycin combination was administered using an age step-down approach, with participants stratified into adults (18–65 years), adolescents (11–17 years) and children (6 months–10 years) presenting with uncomplicated *P. falciparum* malaria. Inclusion criteria required patients to have microscopically confirmed *P. falciparum* mono-infection, with a parasitemia range of 1000 to 100,000 asexual parasites per microliter of blood, along with a history of fever within the previous 24 h. Patients presenting with severe malaria according to the WHO definition, and those with hemoglobin levels below 8 g/dL, were excluded. Additionally, patients with significant medical disorders or those who had received antimalarial treatment in the past 6 weeks were not eligible for inclusion in the study. The clinical trial was approved by the relevant Independent Ethics Committees, national Institutional Review Boards, and local regulatory authorities. The study protocol was registered online prior to start of recruitment into this clinical trial (pactr.samrc.ac.za, PACTR202008909968293).

### Drug administration

Patients were treated with an oral combination therapy consisting of artesunate, fosmidomycin, and clindamycin, administered every 12 h for three days, totaling six doses. The dosing was weight-based, with patients receiving artesunate at 2 mg/kg, fosmidomycin at 30 mg/kg, and clindamycin at 10 mg/kg. The dosages were adjusted to the closest possible match using the available formulations. Artesunate was obtained in 25 mg and 50 mg doses from RenaClinical Ltd (Horley, UK). Fosmidomycin was purchased in 75 mg, 225 mg, and 450 mg capsules from Nextpharma (Göttingen, Germany). Clindamycin (Ratiopharm, Ulm, Germany) was sourced commercially in 150 mg, 300 mg, and 600 mg doses from a community pharmacy (Bavaria-Apotheke, Hamburg, Germany).

### Sample collection and analysis

Up to 9 blood samples were collected on 4 different days (2 mL in heparin tubes). To reduce the times young patients needed to give blood samples a sampling scheme was devised which dependent on the weight of the patient. Samples from patients above 35 kg were taken at 0.25,0.5,0.75,1.5,3,5,8 h (day 0), 24 h (day 1), 48 h (day 2), and at day 7. Patients between 20 and 35 kg had samples taken at 0,1,5,8 h (day 0), 24 h (day 1), 48 h (day 2), and day 7. While patients below 20 kg were sampled at 0,1,8 h (day 0), 24 h (day q1), 48 h (day 2), and day 7. Samples collected concurrently with drug administration were obtained prior to the administration of the medication. Following collection, blood samples were centrifuged for 10 min at 1300 g, and the resulting plasma was stored at − 80 °C until analysis. All samples were shipped from the study site tothe laboratory in Hamburg, Germany on dry ice, with constant temperature monitoring to ensure stability. Plasma concentrations of the medications were measured using liquid chromatography/tandem mass spectrometry (LC–MS/MS). Two distinct methods were previously developed, validated and available for the analysis of fosmidomycin and clindamycin, with calibration ranges established at 0.25–15 mg/L for fosmidomycin and 0.005–0.5 mg/L for clindamycin [11, 12]. For fosmidomycin, samples underwent protein precipitation with 10% trichloroacetic acid with direct injection of the supernatant, while clindamycin samples were treated with acetonitrile for protein precipitation. The supernatants of clindamycin samples were then evaporated to dryness and reconstituted in a 50/50 mixture of methanol and 20 mM ammonium formate. Any samples exceeding the calibration range for clindamycin were appropriately diluted to fall within the established calibration limits.

### Population pharmacokinetic modelling

The concentration–time profiles of fosmidomycin and clindamycin were modeled using non-linear mixed effects modeling in NONMEM^®^ 7.5 (ICON plc, Dublin, Ireland), with Pearl-Speaks-NONMEM (PsN, version 5.24.1). Post-processing was performed using the Xpose (version 4.7.2) and tidyvpc (version 1.5.0) packages in R (version 4.3.2; The R Foundation for Statistical Computing).

#### Structural model selection

Model development was carried out in a step-wise manner. The Laplacian with interaction estimation method was utilized throughout the model-building process. Model selection for nested models was based on the NONMEM objective function (− 2 log-likelihood), where a decrease of 3.84, as derived from the chi-squared distribution (p < 0.05 for one degree of freedom), was considered statistically significant. For non-nested models the Akaike information criterion (AIC) was used. Models with a lower AIC were consider superior. Each compound was modeled separately.

#### Residual variability model

Multiple error models were tested to describe the residual variability. Proportional, additive, and combined error models were evaluated. The combined error model was found to best describe the residual variability and was implemented as follows:

The combined error model was found to best describe the residual error and was implemented as follows:

$$Y = IPRED \cdot \left( {1 + \epsilon_{prop} } \right) + \epsilon_{add}$$

where $Y$ represents the observed concentration, $IPRED$ is the individual predicted concentration, $\epsilon_{prop}$ is the proportional error component, and $\epsilon_{add}$ is the additive error component. Both $\epsilon_{prop}$ and $\epsilon_{add}$ are assumed to be normally distributed with a mean of zero and respective variances.

#### Inter-individual variability model

Pharmacokinetic parameters were assumed to be log-normally distributed and incorporated as follows:

$${\uptheta }_{i} = {\uptheta }_{TV} \cdot e^{{{\upeta }_{{i,{\uptheta }}} }}$$

where $\theta_{i}$ denotes the estimate of the pharmacokinetic parameter for the *i*th individual, $\theta_{TV}$ the typical value or geometric mean of the population, and ${\upeta }_{{i,{\uptheta }}}$ is the inter-individual variability of the parameter ${\uptheta }$ for the *i*th individual.

#### Covariate model building

For both models, allometric scaling with fixed exponents was initially tested for relevant pharmacokinetic parameters (e.g., clearance scaled by body weight to the power of 0.75, volume of distribution scaled by body weight to the power of 1). Subsequently, all other measured covariates (provided in S1) were inspected visually for potential relationships with individual PK parameter estimates. Biologically plausible, as well as promising candidate covariates identified from the visual inspection were then formally tested using the stepwise covariate model (SCM) method within PsN. A decrease in the objective function value (OFV) of 3.84 (p < 0.05) for forward addition and an increase of 6.63 (p < 0.01) for backward elimination were considered statistically significant for inclusion and retention, respectively.

#### Model evaluation

In addition, model evaluation was supported by goodness-of-fit plots and visual predictive checks. The condition number was inspected to assess parameter identifiability and potential collinearity. Shrinkage values for both inter-individual and residual variability were calculated to evaluate the reliability and usability of the goodness-of-fit plots. To obtain robust estimates of parameter uncertainty, the Sampling Importance Resampling (SIR) method [13] was employed.

##### VPC

To account for variability resulting from the different dosing levels, the reference-corrected vpc as described by Ibrahim et al. [14] was used. The reference dataset was set to the median weight of the study population of 29.05 kg, a body temperature of 37.1 °C, and a dose of 900 mg for fosmidomycin. For clindamycin, the reference dataset was set to a weight of 29.05 kg and a dose of 450 mg.

##### SIR

The residual squared error (RSE) as estimated by the covariance step was used for the proposal distribution. Samples were set at 2000, 4000 and resamples at 1000, 2000. The objective function distribution of the proposal distribution should be below the chi-squared distribution to insure accurate results for parameter uncertainty.

#### Handling of data below limit of quantification

Data below the limit of quantification were handled using the M3 method [15].

## Results

### Study population

A total of 40 patients, both male and female, presenting with acute uncomplicated *P. falciparum* malaria were enrolled in the study. The patients were recruited in three age groups: 20 patients aged between 6 months and 10 years, 10 patients aged between 11 and 17 years, and 10 patients aged between 18 and 65 years. A comprehensive summary of the demographic, clinical, and laboratory characteristics of the study population is provided in Table 1 while the complete data is shown in the supplement (S1).

**Table 1:** Demographic and admission laboratory and clinical data of the study population collected during the screening process

	Overall (N = 40)
*Sex*	
Female	17 (42.5%)
Male	23 (57.5%)
*Age (years)*	
Mean (SD)	14.7 (11.6)
Median [Min, Max]	10.8 [3.50, 57.2]
*Weight (kg)*	
Mean (SD)	37.5 (20.2)
Median [Min, Max]	29.1 [12.0, 86.0]
*Body temperature (°C)*	
Mean (SD)	37.1 (1.06)
Median [Min, Max]	36.8 [35.4, 39.0]
*GFR*^a^ (mL/min/1.73 m^*2*^*)*	
Mean (SD)	126 (34.9)
Median [Min, Max]	114 [43.6, 214]
*Hematocrit (%)*	
Mean (SD)	32.7 (3.96)
Median [Min, Max]	32.6 [25.1, 39.9]
*Haemoglobin (g/dL)*	
Mean (SD)	10.9 (1.39)
Median [Min, Max]	10.9 [8.40, 13.9]
*Albumin (g/L)*	
Mean (SD)	36.0 (5.49)
Median [Min, Max]	36.2 [25.5, 54.4]

^a^CKD-EPI formula for adults and bedside Schwartz GFR for below 18 years

### Pharmacokinetic models and evaluation

#### Fosmidomycin model

A total of 242 samples were included in the pharmacokinetic analysis of fosmidomycin. The data were best described by a one-compartment model with linear elimination and first-order absorption, including an absorption lag time. Incorporation of allometric scaling further improved model performance and was therefore included in the final model. Substantial inter-individual variability (IIV) was observed, with the absorption rate constant (*k*_*a*_) and bioavailability (*F*) exhibiting coefficients of variation of 68.4% and 34.7%, respectively. Additionally, stepwise covariate modeling (SCM) identified body temperature as a significant covariate affecting fosmidomycin clearance. Clearance (*CL*) and volume of distribution (*V*) were parameterized as follows:

$$CL_{i} = CL_{TV} \cdot \left( {\frac{WGT}{{29.05\;{\mathrm{kg}}}}} \right)^{0.75} \cdot { }\frac{BT}{{37.1}}^{{\theta_{TEMP} }} \cdot { }e^{{{\upeta }_{{i,{\mathrm{CL}}}} }}$$

where $CL_{i}$ denotes the individual clearance for the *i*th individual., $CL_{TV}$ represents the typical clearance value for an individual with a body weight (WGT) of 29.05 kg and a body temperature (BT) of 37.1, $\theta_{TEMP}$ denotes the covariate effect of body temperature, and $e^{{{\upeta }_{{i,{\mathrm{CL}}}} }}$ the inter-individual variability in $CL$ for the *i*th individual.

$$V_{i} = V_{TV} \cdot \left( {\frac{WGT}{{29.05\;{\mathrm{kg}}}}} \right)^{1} \cdot { }e^{{{\upeta }_{{i,{\mathrm{V}}}} }}$$

where $V_{i}$ denotes the individual volume for the *i*th individual, $V_{TV}$ the typical value at a WGT of 29.05 kg, and $e^{{{\upeta }_{{i,{\mathrm{V}}}} }}$ the inter-individual variability in $V$ for the *i*th individual.

The final model estimates are shown in Table 2 and the final model is included in the supplement (S2).

**Table 2:** The parameter estimates for the final population pharmacokinetic model for fosmidomycin and clindamycin

Fosmidomycin model				
Parameter	Description	Units	Typical value	95% CI
*Structural parameters*				
*CL/F*	Apparent clearance (for a 29.05 kg person at 37.1 °C)	L/h	58.3	[51.0, 67.8]
*V/F*	Apparent volume of distribution (for a 29.05 kg person)	L	248	[201.9, 302.8]
*k*_*a*_	Absorption rate constant	h⁻^1^	0.698	[0.48, 1.0]
*t*_*lag*_	Absorption lag time	h	0.105	[0.03, 0.17]
*F*	Relative bioavailability	–	1 FIXED	–
*Covariate effects*				
*θ*_*TEMP*_	Power exponent for the effect of body temperature on CL/F	–	6.68	[3.6, 9.7]
*Inter-individual variability (IIV)*				
*ω*_*ka*_	IIV on Absorption Rate Constant	%CV	68.3	[45.8, 102.1]
*ω*_*F*_	IIV on Bioavailability	%CV	34.7	[24.3, 48.8]
*Residual error model*				
*σ*_*prop*_	Proportional error	%CV	35.6	[29.7, 41.9]
*σ*_*add*_	Additive error	mg/L	0.197	[0.15, 0.25]
*Clindamycin model*				
Structural parameters				
* CL/F*	Apparent clearance (for a 29.05 kg person)	L/h	8.02	[7.2, 9.0]
* V/F*	Apparent volume of distribution (for a 29.05 kg person)	L	28.4	[25.4, 32.6]
* k*_*a*_	Absorption rate constant	h⁻^1^	2.2	[1.3, 3.8]
* t*_*lag*_	Absorption lag time	h	0.227	[0.20, 0.24]
Inter-individual variability (IIV)				
* ω*_*ka*_	IIV on absorption rate constant	%CV	91.8	[65.2, 129.1]
* ω*_*tlag*_	IIV on absorption lag time	%CV	10.7	[4.9, 20.6]
* ω(k*_*a*_*, t*_*lag*_*)*	Covariance between IIV on ka and tlag	%	− 0.052 (− 63%)	[− 0.11, − 0.013]
Inter-occasion variability (IOV)				
* κ*_*CL/F*_	IOV on apparent clearance	%CV	28.8	[23.7, 34.5]
Residual error model				
* σ*_*prop*_	Proportional error	%CV	32.9	[27.5.0, 39.2]
* σ*_*add*_	Additive error	mg/L	0.0041	[0.003, 0.006]

#### Clindamycin model

A total of 274 samples were included in the pharmacokinetic analysis of clindamycin. The data were best described by a one-compartment model with linear elimination and first-order absorption, including a lag time. Incorporation of allometric scaling further improved the model fit for clindamycin. The largest inter-individual variability (IIV) was observed for the absorption rate constant (ka) with a coefficient of variation of 91.8%. The lag time also demonstrated substantial IIV (10.7% CV), which was found to be correlated with the variability in the absorption rate constant. Additionally, incorporating inter-occasion variability (IOV) on clearance significantly improved model performance. Model estimates are presented in Table 2, and details of the final model are provided in the supplementary material. Aside from body weight, no other significant covariates were identified during the stepwise covariate modeling (SCM) process. Clearance (CL) and volume of distribution (V) were parameterized as follows:

$$CL_{i} = CL_{TV} \cdot \left( {\frac{WGT}{{29.05\;{\mathrm{kg}}}}} \right)^{0.75} \cdot { }e^{{{\upeta }_{{i,{\mathrm{CL}}}} }}$$

where $CL_{i}$ denotes the individual clearance for the *i*th individual., $CL_{TV}$ represents the typical clearance value for an individual with a body weight (WGT) of 29.05 kg, and $e^{{{\upeta }_{{i,{\mathrm{CL}}}} }}$ the inter-individual variability in $CL$ for the *i*th individual.

$$V_{i} = V_{TV} \cdot \left( {\frac{WGT}{{29.05\;{\mathrm{kg}}}}} \right)^{1} \cdot { }e^{{{\upeta }_{{i,{\mathrm{V}}}} }}$$

where $V_{i}$ denotes the individual volume for the *i*th individual, $V_{TV}$ the typical value at a WGT of 29.05 kg, and $e^{{{\upeta }_{{i,{\mathrm{V}}}} }}$ the inter-individual variability in $V$ for the *i*th individual.

The final model estimates are shown in Table 2 and the final model is included in the supplement (S3).

#### Model evaluation

**Fig. 1:**
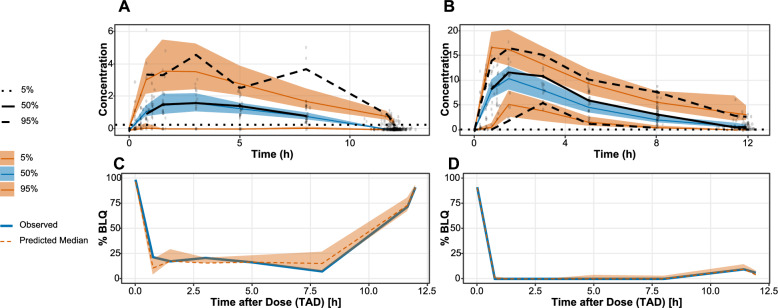
Reference-corrected visual predictive checks (VPCs) for the fosmidomycin (**A**) and clindamycin (**B**) models. The black lines indicate the observed data percentiles at the 5th, 50th, and 95th. The blue lines represent the median (50th percentile) of the simulated data, with the shaded ribbon depicting the 90% confidence interval (CI). The orange lines show the 5th and 95th percentiles of the simulated data, with the shaded area representing the 95% CI. The dotted black line denotes the lower limit of quantification (LLOQ). The second row illustrates the proportion of samples below the LLOQ for fosmidomycin (**C**) and clindamycin (**D**). The blue line indicates the observed fraction of samples below the LLOQ, while the orange lines depict the simulated fraction with the shaded ribbon representing the 90% CI. A total of 1000 samples were used

The GOF plots indicate that the models adequately describe the PK of fosmidomycin and clindamycin and are shown in the supplement (S4, S5). The reference-corrected VPCs showed that the variability was also adequately captured and are shown in Fig. 1.

#### Exposure analysis

**Fig. 2:**
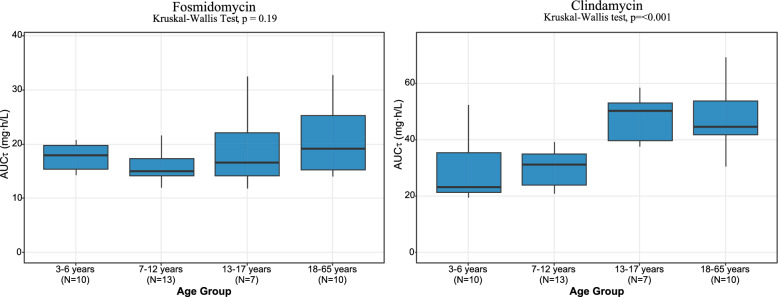
Box-plots showing the AUC_τ_ distribution of fosmidomycin (30 mg/kg) and clindamycin (10 mg/kg) in different age groups.

The final pharmacokinetic models were utilized to calculate the average area under the curve over a dosing interval (AUCτ), derived by taking the AUC over 12 h following the last dose and dividing it by the six dosing intervals. This approach assessed whether the recommended weight-based dosing regimens for fosmidomycin (30 mg/kg) and clindamycin (10 mg/kg) resulted in comparable drug exposure between children and adults. To investigate potential age-related differences, the study population was stratified into four age groups: 3–6 years, 7–12 years, 13–17 years, and 18–65 years. To compare the differences in means of exposure across groups, normality and homoscedasticity were assessed using the Shapiro–Wilk test and Levene’s test, respectively. Because the normality assumption was violated for clindamycin and fosmidomycin in the 3–6 years age group, the Kruskal–Wallis test was employed to evaluate differences in exposure among the groups. The results are presented in Fig. 2. For clindamycin, a Dunn’s post-hoc test with Bonferroni correction was conducted to perform pairwise comparisons between groups [16]. The detailed results of these pairwise tests are provided in the Supplement (S6). To ensure that findings were not influenced by the limited sample size, the original dataset of the study population was used to perform 1,000 simulations, and the resulting simulated exposures were compared to those observed in the actual dataset. These results are illustrated in the supplement (S7).

#### Clindamycin dosing simulation

**Fig. 3:**
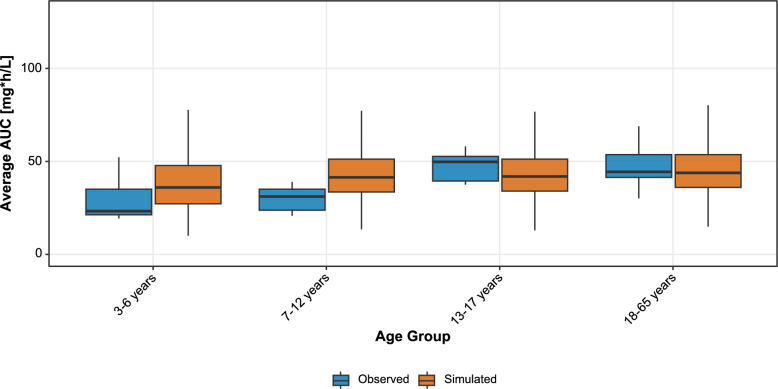
Boxplots illustrating the distribution of AUCτ for clindamycin across different age groups. The blue boxes represent observed values obtained with the original 10 mg/kg dosing scheme. The orange boxes depict AUCτ values under the new dosing scheme, where patients weighing less than 35 kg receive 12 mg/kg, and those above 35 kg continue to receive 10 mg/kg

Dosing simulations were conducted using a virtual patient cohort. For each age group, 1000 virtual patients were created by sampling body weights from a normal distribution, using the age-specific means and standard deviations observed in the study population. Weight-based dosing was applied using a stepwise regimen, with dose reductions at predefined weight cut-offs of 20, 35, and 50 kg, which correspond to the upper limits of the weight ranges for each age group. To achieve equal drug exposure across all weight groups, a dose of 12 mg/kg was selected for patients weighing less than 35 kg, and 10 mg/kg for those above 35 kg. The exact dosing regimen for each weight range is provided in the Supplementary Material (S8). The AUC distribution of the dosing simulation is shown in Fig. 3.

## Discussion

This paper presents a population pharmacokinetic analysis of fosmidomycin and clindamycin, administered with artesunate, in 40 patients with uncomplicated malaria in Gabon. The study data was used to develop robust pharmacokinetic models that characterized the drugs' behavior and revealed important insights into dosing requirements for different age groups. Our findings indicate that while the weight-based dosing for fosmidomycin provides consistent exposure across age groups, the standard regimen for clindamycin results in lower exposure in young children.

The pharmacokinetics of fosmidomycin in our study was best described by a one-compartment model with linear elimination and first-order absorption. While limited data exist on the pharmacokinetics of fosmidomycin in the literature, previous studies have also demonstrated that a one-compartment model best characterizes its pharmacokinetics following oral administration [17–19]. In the presented study, the observed half-life of approximately 2.9 h was between previously reported values. Slightly shorter than the 3.5 h reported by Na-Bangchang et al. and slightly longer than the 2.2 h reported by Ruangweerayut et al., both in studies of fosmidomycin co-administered with clindamycin for *P. falciparum* malaria treatment [18, 19]. Furthermore, the earlier study conducted in cases of uncomplicated *P. falciparum* malaria by Na-Bangchang et al. reported an apparent volume of distribution and apparent clearance rates of 116 L and 26 L/h respectively, while the estimates of the presented study were 248 L and 58.3 L/h were significantly higher. However, the results of Ruangweerayut et al. align more closely with the present study, with an apparent volume of distribution of 174 L and an apparent clearance of 68.3 L/h reported. An interesting finding from Na-Bangchang et al. is that fosmidomycin administered as monotherapy at 1200 mg every 8 h resulted in approximately 30% lower exposure than the combination with clindamycin at 900 mg every 12 h. A drug interaction affecting metabolism or disposition is unlikely. Fosmidomycin is exclusively cleared renally, while clindamycin undergoes primarily hepatic elimination (90%) mainly via CYP3A4 metabolism [10, 20, 21]. Furthermore, clindamycin exhibits extensive protein binding of approximately 85%, whereas fosmidomycin shows minimal protein binding of only 1% [10, 22]. Another possibility may include non-linearity in the drug's bioavailability, which has been described previously by Kuemmerle et al. [10]. However, it is important to note Ruangweerayut et al. observed a linear increase in exposure when the fosmidomycin dose was split into four times daily instead of twice daily [19]. This finding would not be consistent with non-linear bioavailability at higher fosmidomycin doses. Additionally a bias in the assay might lead to these results.

It is important to note that the populations studied differ significantly: the previous studies in Thai adults and the current research involving both children and adults in Gabon. Additionally, the quantification of fosmidomycin was performed using a bioassay based on the observation of growth inhibition zones at varying fosmidomycin concentrations, rather than the LC–MS/MS method employed in this study. This was the case for Ruangweerayut et al. and Na-Bangchang et al. This approach introduces the potential for interference from clinical blood samples, such as co-medications and active metabolites, which could lead to biased or inaccurate quantification using the bioassay. The assay was never compared to a different assay-type such as LC–MS/MS making the comparison difficult. These differences in study populations, dosing regimens, bioanalytical assay, and co-administered medications could contribute to the observed discrepancies in pharmacokinetic parameters. Furthermore, we found that body temperature appears to influence the clearance of fosmidomycin. This could be a critical factor, especially in cases of severe malaria where high fever is common. This finding is significant and warrants further investigation, especially concerning the pharmacokinetics and dosing of fosmidomycin in patients with severe malaria and elevated body temperatures. Our model predicts a 60% increase in clearance at a body temperature of 40 °C, highlighting the potential impact of fever on drug disposition in severe cases. It is important to note that temperature measurements were collected only twice a day. As fever is a dynamic process, particularly in malaria, these discrete measurements do not capture the complete temporal profile of temperature fluctuations. Therefore, the observed temperature effect should be interpreted cautiously, especially when extrapolating to body temperature values outside the range measured in this study. Despite this limitation, the covariate was retained in the model due to the significant improvement in model performance, and the authors believe that the impact of febrile episodes on drug pharmacokinetics warrants further investigation in future research.

The pharmacokinetics of clindamycin in our study were also best described by a one-compartment model, which aligns with previous literature [18, 23–26]. The parameter estimates obtained in our analysis were consistent with those reported in earlier studies. In our study, we observed a half-life of approximately 2.5 h, slightly shorter than the roughly 3 h reported in most previous research. We employed allometric scaling based on body weight with fixed exponents, a method also described by Smith et al. [23]. Additionally, Bouazza et al. identified body weight as a significant covariate on clearance [24]. Smith et al. further reported that albumin levels influenced the volume of distribution of clindamycin; however, in our analysis, we did not observe a significant effect of albumin on pharmacokinetic parameters. The measured exposures of clindamycin in our study were comparable to those reported by Smith et al. and Hornik et al., with median exposures between 40 and 50 mg·h/L following intravenous administration, which aligns with our median exposure of approximately 30–40 mg·h/L. Assuming a bioavailability of around 88% it also is similar to the 32 mg/L·h found by Na-Bangchang [18, 23, 25].

An important difference between previous studies examining the fosmidomycin-clindamycin combination against falciparum malaria is the addition of artesunate in the current study. This raises the possibility of drug-drug interactions. As the presented study did not include a study arm without artesunate, the potential for drug-drug interactions could not be directly investigated. An interaction affecting fosmidomycin seems unlikely given its renal elimination pathway. One study indicated that artesunate and dihydroartemisinin might induce CYP3A4, although the observed effect was rather small [27]. This could theoretically lead to decreased clindamycin exposure through enhanced metabolism. However, when comparing clindamycin exposures in the current study to those reported in previous *P. falciparum* malaria studies using similar dosing regimens, comparable exposures were achieved [18, 19], suggesting that any such effect, if present, is most likely not clinically relevant. A key finding of our analysis is that clindamycin exposure is significantly lower in children under 12 years of age. This finding is consistent with previous studies showing that children require higher weight-based doses to achieve similar exposures as adults: 12 mg/kg for children aged 2–6 years, 10 mg/kg for those aged 6–12 years, compared to approximately 8.5 mg/kg in adults [23]. Given that previous literature reports a safe and effective exposure range of approximately 40–50 mg·h/L, we used the higher exposure levels observed in adults as a target for the younger age groups. Dose simulation suggested that increasing the dose to 12 mg/kg for children weighing less than 35 kg would achieve more consistent and adequate drug exposure across different age groups. Research has demonstrated that clindamycin exhibits antimalarial activity at doses as low as 5 mg/kg, which could argue against higher doses to avoid potentially increasing adverse drug effects [28]. However, the rationale for antimalarial combination therapy extends beyond malaria treatment alone. In endemic settings, studies have suggested that bacterial infections are frequently misdiagnosed as malaria, and combination therapies may offer dual antimalarial and antibacterial coverage [29]. Therefore, dosing regimens should aim to achieve exposure levels that have demonstrated efficacy in antibacterial treatment, potentially providing benefit for both correctly diagnosed malaria cases and misdiagnosed bacterial infections.

A notable limitation of this analysis is the difficulty in interpreting the relationship between exposure and therapeutic outcome. In the study, the artesunate–fosmidomycin–clindamycin combination achieved a 96% cure rate [30], which precludes establishing a correlation between lower exposure and treatment failure. Additionally, the study included only 40 patients, with particularly sparse sampling data in the younger children. Furthermore, the study was conducted at a single site, resulting in a relatively homogeneous study population, which may limit the generalizability of the findings.

## Conclusion

We developed robust population pharmacokinetic models for both fosmidomycin and clindamycin. The analyses demonstrated that body weight influences both the volume of distribution and clearance for each drug. Additionally, body temperature was found to affect fosmidomycin clearance. Fosmidomycin exposures were similar across different age groups, suggesting consistent pharmacokinetics, whereas clindamycin exposure was notably lower in children under 12 years of age. Simulation results indicated that increasing the dose to 12 mg/kg for children weighing less than 35 kg could help address this discrepancy and achieve more uniform drug exposure across age groups.

## Supplementary Information

Additional file1 (DOCX 706 kb)

## Data Availability

The data that support the findings of this study are available from the corresponding author, [SW], upon reasonable request.
