# Supplementary material for: Population pharmacokinetics of fosmidomycin and clindamycin in combination with artesunate for uncomplicated Plasmodium falciparum malaria in Gabonese children and adults
Source: Malar J. 2026 Mar 19;25:152. doi: 10.1186/s12936-026-05872-6 (PMC13063642; doi:10.1186/s12936-026-05872-6)
Supplement: Supplementary file 1 — Additional file1 (DOCX 706 kb) [file 12936_2026_5872_MOESM1_ESM.docx]

**S 1** Demographic and admission laboratory and clinical data of the study population

|  | **Overall (N=40)** |
| --- | --- |
| ᵃCKD-EPI formula for Adults and Bedside Schwartz GFR for below 18 Years | |
| **Sex** |  |
| Female | 17 (42.5%) |
| Male | 23 (57.5%) |
| **Age (year)** |  |
| Mean (SD) | 14.7 (11.6) |
| Median [Min, Max] | 10.8 [3.50, 57.2] |
| **Weight (kg)** |  |
| Mean (SD) | 37.5 (20.2) |
| Median [Min, Max] | 29.1 [12.0, 86.0] |
| **Height (cm)** |  |
| Mean (SD) | 140 (24.0) |
| Median [Min, Max] | 140 [98.0, 182] |
| **Body Temperature (°C)** |  |
| Mean (SD) | 37.1 (1.06) |
| Median [Min, Max] | 36.8 [35.4, 39.0] |
| **Total Bilirubin (mg/dL)** |  |
| Mean (SD) | 0.785 (0.455) |
| Median [Min, Max] | 0.673 [0.199, 2.15] |
| **Direct Bilirubin (mg/dL)** |  |
| Mean (SD) | 0.259 (0.140) |
| Median [Min, Max] | 0.254 [0.0351, 0.766] |
| **Glucose (mg/dL)** |  |
| Mean (SD) | 85.5 (20.5) |
| Median [Min, Max] | 85.3 [30.6, 160] |
| **Albumin (g/L)** |  |
| Mean (SD) | 36.0 (5.49) |
| Median [Min, Max] | 36.2 [25.5, 54.4] |
| **Urea (mg/mL)** |  |
| Mean (SD) | 19.5 (6.03) |
| Median [Min, Max] | 19.0 [10.2, 39.0] |
| **Serum Creatinine (mg/mL)** |  |
| Mean (SD) | 0.574 (0.219) |
| Median [Min, Max] | 0.558 [0.261, 1.22] |
| **GFRᵃ (mL/min/1.73 m²)** |  |
| Mean (SD) | 126 (34.9) |
| Median [Min, Max] | 114 [43.6, 214] |
| **Alanine Aminotransferase (U/L)** |  |
| Mean (SD) | 16.0 (13.5) |
| Median [Min, Max] | 13.1 [5.80, 90.0] |
| **Alkaline Phosphatase (U/L)** |  |
| Mean (SD) | 194 (110) |
| Median [Min, Max] | 186 [32.8, 451] |
| **Aspartate Aminotransferase (U/L)** |  |
| Mean (SD) | 22.4 (7.87) |
| Median [Min, Max] | 21.3 [11.2, 51.7] |
| **Sodium (mmol/L)** |  |
| Mean (SD) | 137 (4.06) |
| Median [Min, Max] | 137 [128, 145] |
| **Potassium (mmol/L)** |  |
| Mean (SD) | 4.17 (0.685) |
| Median [Min, Max] | 4.00 [3.40, 7.30] |
| **Hematocrit (%)** |  |
| Mean (SD) | 32.7 (3.96) |
| Median [Min, Max] | 32.6 [25.1, 39.9] |
| **Haemoglobin (g/dL)** |  |
| Mean (SD) | 10.9 (1.39) |
| Median [Min, Max] | 10.9 [8.40, 13.9] |
| **Red Blood Cell Count (x10^6/mm3)** |  |
| Mean (SD) | 4.26 (0.437) |
| Median [Min, Max] | 4.27 [3.28, 5.11] |
| **White Blood Cell Count (Count x10^3/mm3)** |  |
| Mean (SD) | 7.06 (2.79) |
| Median [Min, Max] | 6.67 [3.40, 18.3] |
| **Platelet Count (Count x10^3/mm3)** |  |
| Mean (SD) | 158 (73.7) |
| Median [Min, Max] | 151 [58.0, 376] |

**S 2** Fosmidomycin final model

$SUBROUTINES ADVAN2 TRANS2

$PK

TVCL = THETA(1) * ((WGT/29.05)**0.75) * ((BT/37.1)**THETA(6))

CL = TVCL

TVV = THETA(2) * (WGT/29.05)**1

V = TVV * EXP(ETA(1))

S2=V

TVKA = THETA (3)

KA = TVKA * EXP(ETA(2))

ALAG1=THETA(4)

TVF1 = THETA(5)

F1 = TVF1 * EXP(ETA(3))

$ERROR

LOQ=0.25

IPRED = F

IRES = DV-IPRED

W = SQRT(SIGMA(1)*IPRED*IPRED + SIGMA(2))

IWRES = IRES/(W)

IF(COMACT==1) PREDV=IPRED

DUM = (LOQ - IPRED) / W

CUMD = PHI(DUM)

TYPE=1

IF(DV<LOQ) TYPE=2

IF(MDV==1) TYPE=0

IF(TYPE.EQ.2) DV_LOQ=LOQ

IF (TYPE .NE. 2.OR.NPDE_MODE==1) THEN

F_FLAG = 0

Y = IPRED + IPRED*EPS(1) + EPS(2)

ENDIF

IF (TYPE .EQ. 2.AND.NPDE_MODE==0) THEN

F_FLAG = 1

Y = CUMD

MDVRES=1

ENDIF

$THETA

(0, 58.3) ;1_CL

(0, 248) ;2_V

(0, 0.698) ;3_KA

(0, 0.105) ;4_ALAG1

(1) FIX ;5_F1

(-100, 6.68,100000) ;6_CLBT1

$OMEGA

0 FIX ; 1_IIV_V

0.383 ; 2_IIV_KA

0.114 ; 3_IIV_F1

$SIGMA

0.127 ;Prop.Err_PG

0.0389 ;add.Err_PG

$EST METHOD=1 INTER LAPLACIAN MAXEVAL=5000 NOABORT SIG=2 PRINT=1

$COV PRINT=E MATRIX=S

S 3 Clindamycin final model

$SUBROUTINES ADVAN2 TRANS2

$PK

IF(EVID.EQ.1.AND.AMT.GT.0) DOSE=AMT

FLAG0 = 0

FLAG1 = 0

FLAG2 = 0

FLAG3 = 0

FLAG4 = 0

FLAG5 = 0

IF(OCC.EQ.1) FLAG1 = 1

IF(OCC.EQ.2) FLAG2 = 1

IF(OCC.EQ.3) FLAG3 = 1

IF(OCC.EQ.4) FLAG4 = 1

IF(OCC.EQ.5) FLAG5 = 1

IF(OCC.EQ.6) FLAG6 = 1

IOV_CL =FLAG1*ETA(6)+FLAG2*ETA(7)+FLAG3*ETA(8)+FLAG4*ETA(9)+FLAG5*ETA(10)+FLAG6*ETA(11)

;----DISTRIBUTION MODEL-------------

TVCL = THETA(1) * (WGT/29.05)**0.75

CL = TVCL*EXP(ETA(1) + IOV_CL)

TVV = THETA(2) * (WGT/29.05)**1

V = TVV*EXP(ETA(2))

S2=V

;--------ABSORPTION MODEL-----------

TVKA = THETA(3)

KA = TVKA*EXP(ETA(4))

TVF1 = THETA(4)

F1 = TVF1*EXP(ETA(3))

TVALAG1 = THETA(5)

ALAG1 = TVALAG1 * EXP(ETA(5))

$ERROR

LOQ=0.005

IPRED = F

IRES = DV-IPRED

W = SQRT(SIGMA(1)*IPRED*IPRED + SIGMA(2))

IWRES = IRES/(W)

IF(COMACT==1) PREDV=IPRED

DUM = (LOQ - IPRED) / W

CUMD = PHI(DUM)

TYPE=1

IF(DV<LOQ) TYPE=2

IF(MDV==1) TYPE=0

IF(TYPE.EQ.2) DV_LOQ=LOQ

IF (TYPE .NE. 2.OR.NPDE_MODE==1) THEN

F_FLAG = 0

Y = IPRED + IPRED*EPS(1) + EPS(2)

ENDIF

IF (TYPE .EQ. 2.AND.NPDE_MODE==0) THEN

F_FLAG = 1

Y = CUMD

MDVRES=1

ENDIF

$THETA

(0.001, 8.02) ;1_CL

(0.001, 28.4) ;2_Vc

(0.001, 2.2) ;3_KA

(1) FIX ;4_F1

(0, 0.227) ;5_LAG-Time

$OMEGA

0 FIX ;1_IIV_CL

0 FIX ;2_IIV_Vc

0 FIX ;3_IIV_F1

$OMEGA BLOCK(2)

0.611 ;4_IIV_KA

-0.0524 0.0114 ;5_IIV_LAG-Time

$OMEGA BLOCK(1) 0.0798 ; IOV for CL

$OMEGA BLOCK(1) SAME ;

$OMEGA BLOCK(1) SAME ;

$OMEGA BLOCK(1) SAME ;

$OMEGA BLOCK(1) SAME ;

$OMEGA BLOCK(1) SAME ;

$SIGMA

0.108 ;prop Error

1.69E-05 ;add Error

$EST METHOD=1 LAPLACIAN MAXEVAL=99999 MCETA=5 SIG=3 PRINT=5 NOABORT POSTHOC INTERACTION

$COV PRINT=E


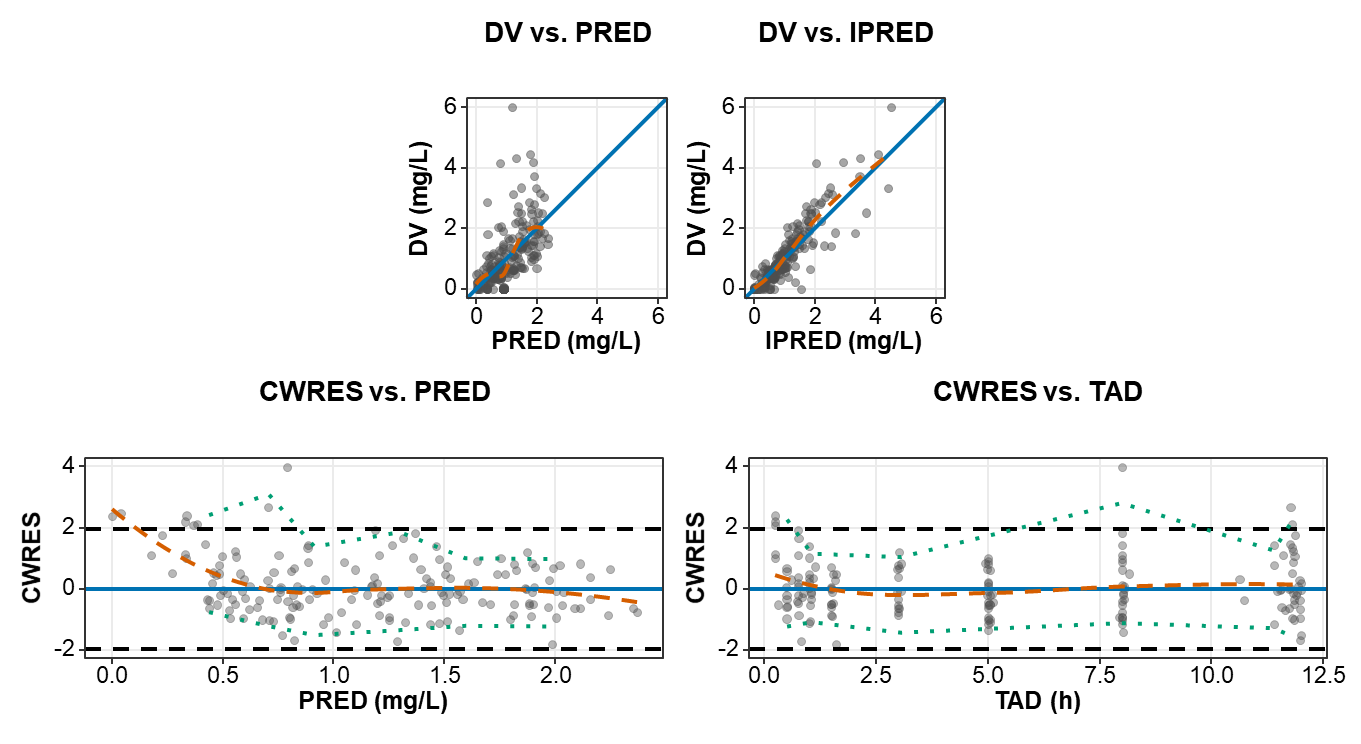


**S 4** GOF Plots for fosmidomycin.


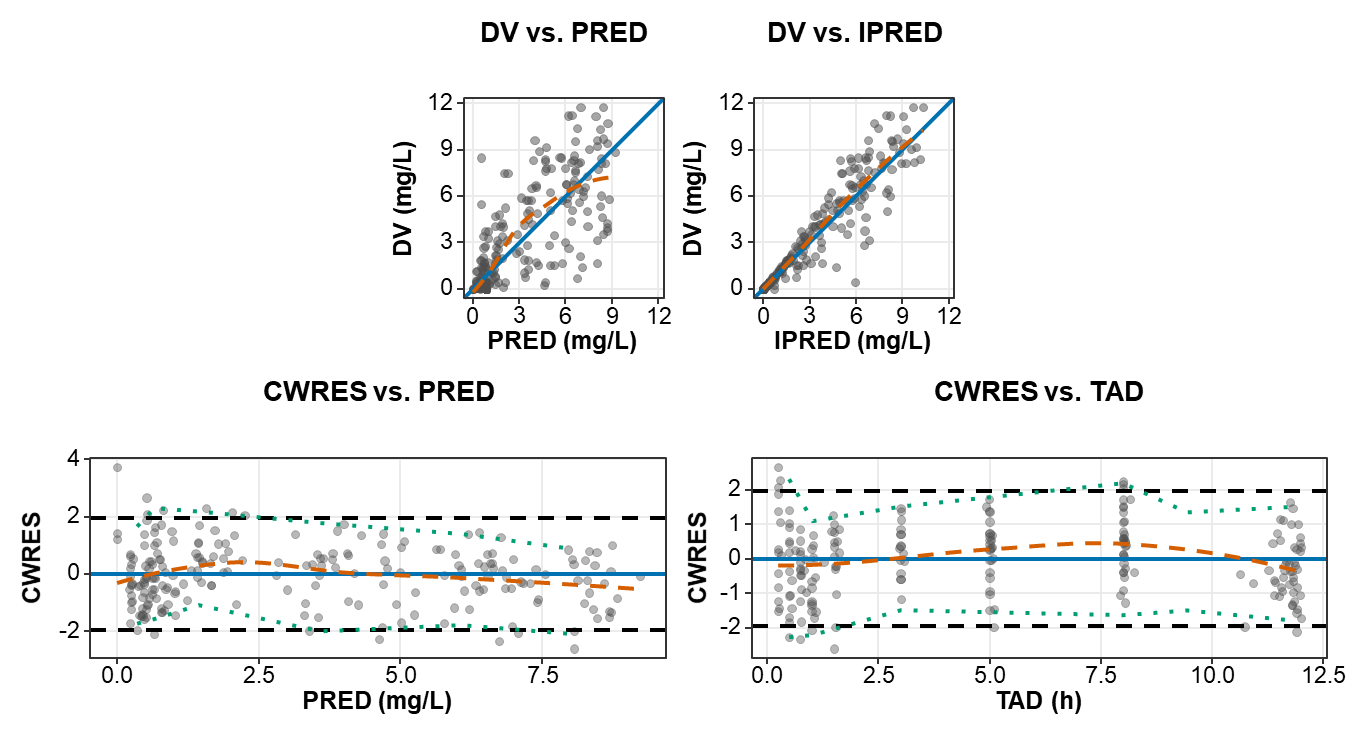


**S 5** GOF Plots for clindamycin.


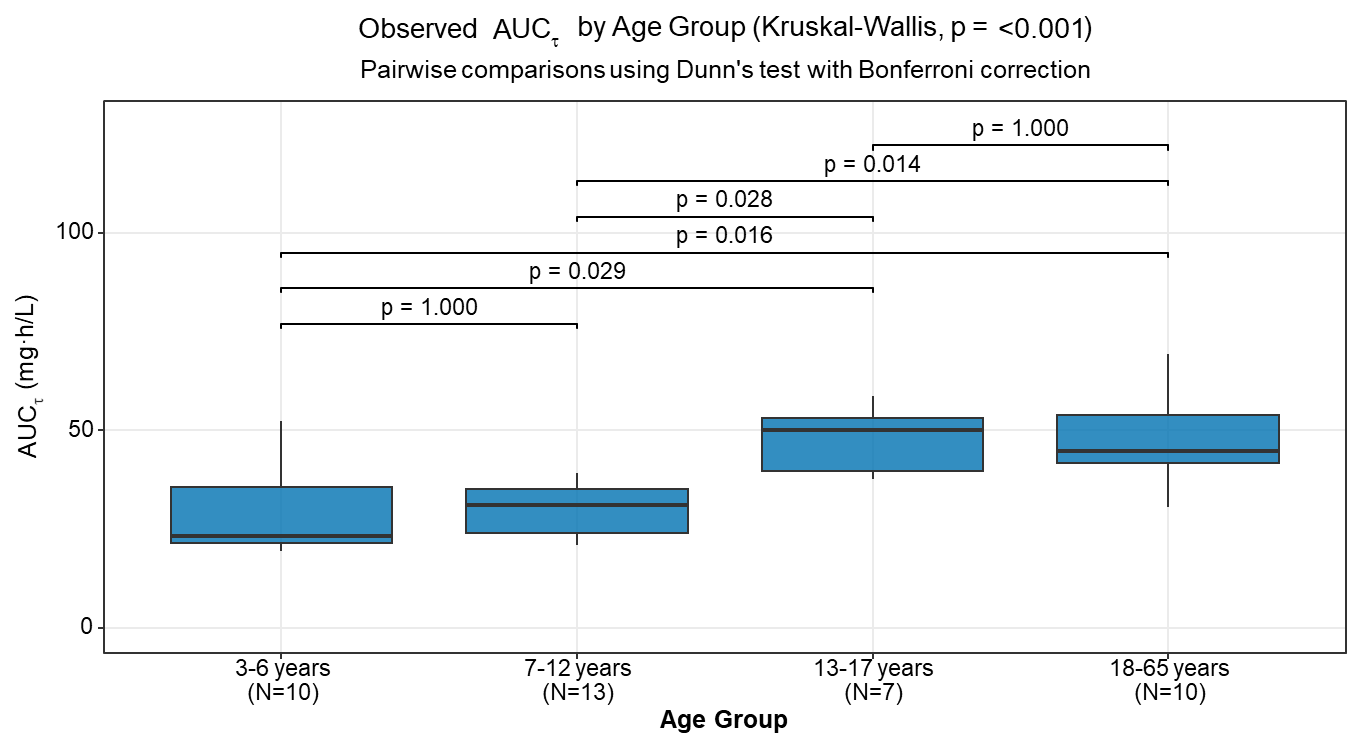


**S 6** Pairwise test of clindamycin exposure in different age groups.


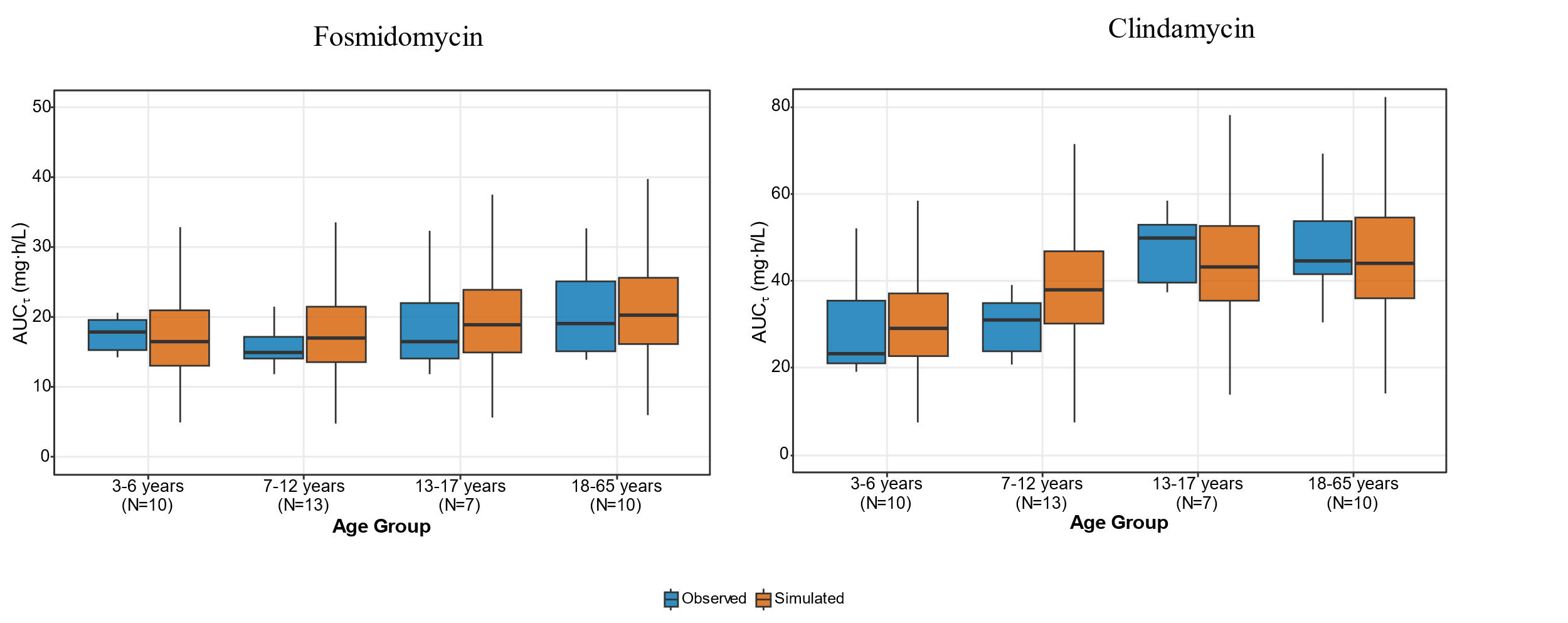


**S 7** Exposure of fosmidomycin and clindamycin in different age groups. The blue boxes show the observed exposures, while the orange boxes show the exposure of 1000 simmulations.

**S 8** Weight ranges for a given dose in the new dosing scheme.

| **Clindamycin Dose [mg]** | **Weight Range [kg]** |
| --- | --- |
| 150 | <18.7 |
| 300 | 18.7-31.2 |
| 450 | 31.2-52.5 |
| 600 | 52.5-67.5 |
| 750 | 67.5-82.5 |
| 900 | 82.5-97.5 |
